# PREPARING THE GROUND: DEVELOPING COMMUNITY-BASED STRATEGIES TO ENGAGE OLDER BLACK MEN IN HEALTH RESEARCH

**DOI:** 10.1093/geroni/igac059.1912

**Published:** 2022-12-20

**Authors:** Jamie Mitchell, Kent Key, Elena Flores, Sean Knurek, Tam Perry, Vanessa Rorai, Joan Ilardo, Alexandra Sass

**Affiliations:** University of Michigan, Ann Arbor, Michigan, United States; Michigan State University, Flint, Michigan, United States; University of Michigan, Ann Arbor, Michigan, United States; Michigan State University, Corunna, Michigan, United States; Wayne State University, Detroit, Michigan, United States; Wayne State University, Detroit, Michigan, United States; Michigan State University, East Lansing, Michigan, United States; University of Michigan, Ann Arbor, Michigan, United States

## Abstract

Black men across the lifespan are overburdened by poor health and underrepresented as participants in health research. The Flint Healthier Black Elders program seeks to engage more older Black men in research that could contribute to health discoveries by developing and testing strategies to recruit Black men into a community participant research pool (PRP). The PRP recruitment strategies account for the influence of gender role norms, mistrust derived from current and historical research and medical abuses, and other factors that affect older Black men’s willingness to participate in safe and ethical research. This initiative also focuses on building trust in research engagement by foregrounding the voices of local older men as community stakeholders and research gatekeepers, and tailoring multimedia recruitment materials to represent older Black men more fully and positively. Videos and print materials developed as recruitment tools specifically tailored to older Black men were pilot tested for messaging and impact, and the results of this community-driven process can serve as an innovative model for equitable and trustworthy research recruitment in Black communities. We would like to acknowledge the contributions of the Flint Healthier Black Elders Community Advisory Board: Yaushica Aubert, Rev. Dr. Sarah Bailey, E. Hill DeLoney, Luther Evans, Ella Greene-Moton, Cynthia Howell, Bishop Bernadel Jefferson, Beverly Lewis, Geraldine Redmond, Sharon Saddler, Arlene Sparks, Erica Thrash-Sall.

